# Maternal Interleukin-6 as a Predictor of Preterm Birth: A Prospective Observational Study at the County Clinical Emergency Hospital of Sibiu

**DOI:** 10.7759/cureus.97732

**Published:** 2025-11-25

**Authors:** Sandra Neamtu, Raluca Badila, Radu Chicea

**Affiliations:** 1 Anesthesia and Critical Care, County Clinical Emergency Hospital of Sibiu, Sibiu, ROU; 2 Obstetrics and Gynecology, Lucian Blaga University of Sibiu, Sibiu, ROU

**Keywords:** biomarkers, cytokines, interleukin-6, intrauterine inflammation, maternal serum, premature rupture of membranes, preterm birth

## Abstract

Background

Preterm birth remains a leading cause of neonatal morbidity and mortality worldwide. Inflammation plays a crucial role in its pathophysiology, with interleukin-6 (IL-6) recognized as a key cytokine mediating preterm labor. This study aimed to evaluate the relationship between maternal serum IL-6 levels and gestational age at delivery, birthweight, and neonatal outcomes in women presenting with spontaneous preterm labor.

Methods

A prospective observational study was conducted at the County Clinical Emergency Hospital of Sibiu between January 2024 and January 2025. Fifty pregnant women between 27 and 34 weeks of gestation were enrolled. Maternal serum IL-6 concentrations were measured using enzyme-linked immunosorbent assay (ELISA). Clinical variables and neonatal outcomes were recorded.

Results

The mean gestational age at delivery was 31.86 ± 1.88 weeks, and the mean maternal IL-6 was 12.76 ± 3.04 pg/mL. IL-6 correlated inversely with both gestational age (r = −0.709, p < 0.001) and birthweight (r = −0.721, p < 0.001). Women delivering before 32 weeks had significantly higher IL-6 concentrations than those delivering later (p < 0.001). Neonates born to mothers with IL-6 ≥ 11 pg/mL had a higher rate of NICU admission (55.6% vs. 14.3%, p = 0.020). Overall, neonatal survival to discharge was 94%.

Conclusions

Maternal IL-6 levels are significantly elevated in women delivering preterm and correlate inversely with gestational age and neonatal birthweight. These findings support the use of IL-6 as a predictive biomarker for preterm birth. Routine IL-6 assessment may improve early identification and management of at-risk pregnancies.

## Introduction

Preterm birth, defined as delivery before 37 completed weeks of gestation, remains a major public health challenge, accounting for nearly 15 million births annually worldwide and contributing to approximately one million neonatal deaths each year [1]. Despite advances in perinatal care, preterm birth continues to be the leading cause of neonatal morbidity and long-term neurodevelopmental impairment. Understanding its underlying mechanisms is therefore a global research priority.

Among the multifactorial pathways leading to preterm labor, intrauterine inflammation has emerged as a principal mechanism [2,3]. Both microbial invasion and sterile inflammatory processes can activate the maternal-fetal immune system, resulting in increased production of cytokines such as interleukin-6 (IL-6), tumor necrosis factor-alpha (TNF-α), and interleukin-1β [4,5]. IL-6, in particular, has been identified as a pivotal mediator linking inflammation to parturition. It promotes cervical ripening, prostaglandin synthesis, and myometrial contractility while also contributing to fetal inflammatory responses [6,7].

Previous studies have demonstrated elevated IL-6 concentrations in maternal serum, amniotic fluid, and cervicovaginal secretions among women who subsequently delivered preterm [8-10]. Furthermore, IL-6 elevation is observed in both microbial invasion of the amniotic cavity and sterile intrauterine inflammation, supporting its role as a sensitive biomarker for intrauterine immune activation [11-13]. Goldenberg et al. further emphasized the connection between intrauterine infection and preterm delivery [14], while more recent work by Kacerovsky and Jacobsson demonstrated that IL-6-driven cytokine signaling contributes to both infectious and sterile inflammation associated with preterm labor [15].

However, while the diagnostic and prognostic potential of IL-6 has been widely studied in high-resource research settings, limited data are available from Central and Eastern European populations, where biological, environmental, and healthcare factors may differ. Furthermore, the utility of maternal serum IL-6 as a noninvasive biomarker for early prediction of preterm delivery remains under-evaluated in routine obstetric practice.

Therefore, the present study aimed to investigate the association between maternal serum IL-6 concentrations and gestational age at delivery, birthweight, and neonatal outcomes among women presenting with spontaneous preterm labor at the County Clinical Emergency Hospital of Sibiu. By assessing IL-6 as a potential predictive biomarker, this research seeks to enhance early identification of high-risk pregnancies and to contribute to improved perinatal management strategies.

## Materials and methods

Study design and setting

This investigation was designed as a prospective, single-center observational study carried out in the Department of Obstetrics and Gynecology at the County Clinical Emergency Hospital of Sibiu, Romania. The institution serves as a tertiary referral and perinatal care center, providing comprehensive obstetric and neonatal services for both high- and moderate-risk pregnancies. Data collection and clinical monitoring were performed over a 12-month period from January 2024 to January 2025, encompassing all eligible admissions for spontaneous preterm labor within the specified gestational interval.

Study population

A total of 50 pregnant women, aged 18 to 40 years, were prospectively enrolled following admission for spontaneous preterm labor between 27 + 0 and 34 + 6 weeks of gestation. Eligible participants were those with confirmed singleton pregnancies, viable fetuses, and spontaneous onset of preterm labor, defined as regular uterine contractions accompanied by progressive cervical dilation and effacement, with or without preterm premature rupture of membranes (PPROM). Patients were excluded if they presented with multiple gestations, major structural or chromosomal fetal anomalies, or confirmed intrauterine fetal demise at the time of admission. All participants provided written informed consent prior to inclusion in the study.

Clinical assessment

On admission, each participant underwent a detailed clinical and obstetric examination. Gestational age was confirmed by first-trimester ultrasonography or last menstrual period if early scans were unavailable. Fetal presentation, membrane status (intact or ruptured), and maternal comorbidities such as preeclampsia, oligohydramnios, thrombophilia, placenta praevia, and hypothyroidism were recorded.

Sample collection and laboratory analysis

Venous blood samples were collected at admission, prior to administration of corticosteroids or tocolytic therapy. Serum IL-6 levels were measured using a quantitative enzyme-linked immunosorbent assay (ELISA). A concentration of ≥11 pg/mL was used to define elevated IL-6, consistent with published reference thresholds [9]. Primary outcomes were gestational age at delivery and neonatal birthweight. Secondary outcomes included neonatal intensive care unit (NICU) admission and survival to discharge.

Both descriptive and inferential statistical methods were used in the analysis. Descriptive statistics were applied to summarize maternal and neonatal characteristics. Continuous variables such as gestational age, birthweight, and maternal IL-6 concentrations were expressed as mean ± standard deviation (SD), while categorical variables were reported as frequencies (n) and percentages (%).

Inferential statistics were applied to evaluate relationships between clinical and biochemical parameters. Group comparisons between early preterm (<32 weeks) and late preterm (≥32 weeks) deliveries were performed using the Welch’s t-test for continuous data, and the chi-square (χ²) or Fisher’s exact test for categorical data, as appropriate. The Pearson correlation coefficient (*r*) was used to assess linear associations between maternal IL-6 levels, gestational age, and neonatal birthweight. A p-value < 0.05 was considered statistically significant. Statistical analyses were performed using IBM SPSS Statistics for Windows, Version 26 (Released 2018; IBM Corp., Armonk, New York, United States).

Statistical analysis

Continuous variables such as gestational age, birthweight, and maternal IL-6 concentration were summarized as mean values with standard deviations, while categorical data were described as frequencies and percentages. For comparison, women who delivered before 32 weeks of gestation were evaluated against those delivering later in pregnancy. The relationships between maternal IL-6 levels, gestational age, and neonatal birthweight were examined to identify potential clinical correlations. The occurrence of NICU admission was further analyzed according to maternal IL-6 concentration categories. A p-value below 0.05 was considered indicative of a statistically meaningful association.

## Results

A total of 50 women with spontaneous preterm labor between 27 + 0 and 34 + 6 weeks of gestation were included in the analysis. The mean gestational age at delivery was 31.86 ± 1.88 weeks, and the mean maternal serum IL-6 concentration was 12.76 ± 3.04 pg/mL. Most fetuses presented cephalically (n = 43, 86%), followed by breech (n = 5, 10%) and transverse (n = 2, 4%) presentations.

Maternal demographics

Table 1 presents the distribution of maternal age, place of residence, and parity among the study participants. The majority of women were aged 30-39 years (n = 24, 48%), with most residing in rural areas (n = 28, 56%). Nearly half of the participants (n = 23, 46%) were in their first pregnancy, while n = 16 (32%) and n = 11 (22%) were in their second and third or higher pregnancies, respectively. This demographic distribution is consistent with epidemiologic patterns of preterm birth in regional Romanian populations.

**Table 1:** Maternal age, demographic origin, and gestational parity distribution

Variable	Category	n (%)
Age (years)	16-19	4 (8%)
	20-29	18 (36%)
	30-39	24 (48%)
	≥40	4 (8%)
Residence	Rural	28 (56%)
	Urban	22 (44%)
Gravidity	I	23 (46%)
	II	16 (32%)
	≥ III	11 (22%)

Comparison between early and late preterm births

Women who delivered before 32 weeks (n = 20) had significantly higher mean IL-6 levels (14.6 ± 2.9 pg/mL) compared to those who delivered at or after 32 weeks (n = 30; 11.5 ± 2.4 pg/mL). This difference was statistically significant (Welch’s *t*(33.8) = 4.12, p < 0.001, Cohen’s *d* = 1.10), representing a large effect size. These findings indicate that elevated maternal IL-6 levels are strongly associated with earlier delivery.

Maternal inflammatory and laboratory parameters

Table 2 summarizes maternal inflammatory markers and laboratory findings at admission. Elevated IL-6 and C-reactive protein (CRP) levels were observed in the majority of cases, indicating a systemic inflammatory response even in the absence of overt infection. Mild leukocytosis and neutrophilia were frequent findings, while elevated procalcitonin was documented in approximately one-quarter of patients.

**Table 2:** Maternal laboratory and inflammatory parameters

Parameter	Normal range	Mean ± SD	Elevated cases n (%)	Clinical interpretation
Interleukin-6 (pg/mL)	<10.0	12.8 ± 3.0	36 (72%)	Elevated in majority, linked to inflammation
C-reactive protein (mg/L)	<5.0	8.2 ± 2.4	32 (64%)	Suggestive of subclinical infection
Leukocyte count (×10⁹/L)	4.0–10.0	11.6 ± 2.3	28 (56%)	Mild leukocytosis
Neutrophil percentage (%)	45–70	72.5 ± 6.8	30 (60%)	Inflammatory activation
Procalcitonin (ng/mL)	<0.05	0.18 ± 0.09	14 (28%)	Possible bacterial involvement

Correlation analyses

**Figure 1:**
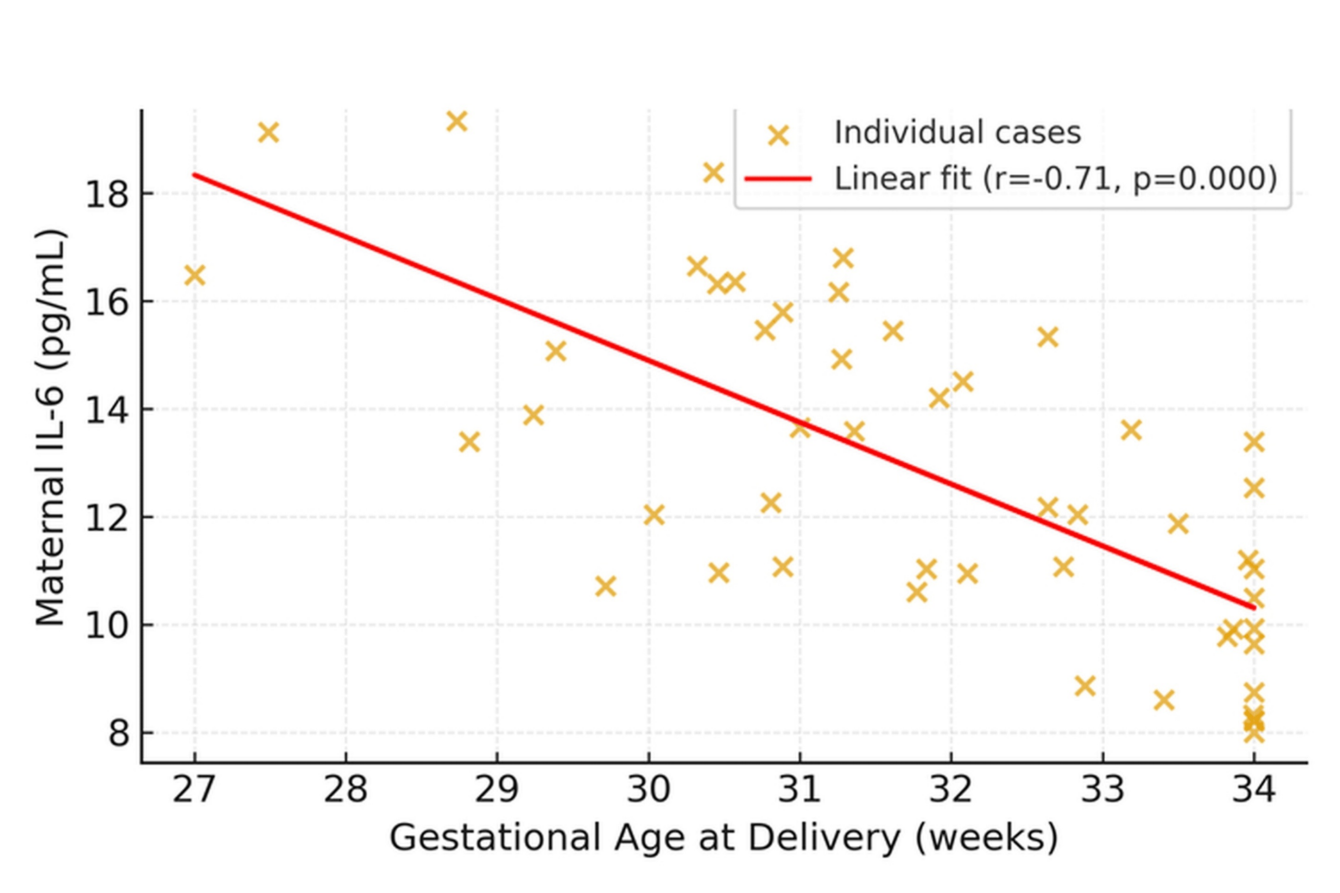
Correlation between maternal IL-6 concentrations and gestational age at delivery

Maternal IL-6 levels showed a strong inverse correlation with gestational age at delivery (*r* = −0.709, p < 0.001) and neonatal birthweight (*r* = −0.721, p < 0.001), as illustrated in Figure 1. These results confirm that higher IL-6 concentrations are associated with earlier delivery and lower fetal growth potential.

Neonatal outcomes

Neonatal characteristics are presented in Table 3. The mean birthweight was 1,700 ± 430 g, consistent with moderate prematurity. The mean Apgar scores were 7.2 ± 1.1 at one minute and 8.4 ± 0.8 at five minutes, reflecting adequate postnatal adaptation in most cases.

**Table 3:** Neonatal outcomes

Parameter	Mean ± SD / n (%)	Range	Clinical interpretation
Birthweight (g)	1,700 ± 430	980-2,480	Moderate preterm profile
Apgar score at 1 min	7.2 ± 1.1	5-9	Lower in IL-6–elevated cases
Apgar score at 5 min	8.4 ± 0.8	6-10	Good recovery overall
NICU admission	22 (44%)	-	Frequent in high-IL-6 group
Neonatal survival to discharge	47 (94%)	-	Comparable to published outcomes

A total of 22 neonates (44%) required admission to the NICU, primarily for respiratory distress or thermal instability. NICU admission was significantly more common among neonates born to mothers with elevated IL-6 levels (≥11 pg/mL; 20/36, 55.6%) than among those with lower IL-6 levels (2/14, 14.3%). This difference was statistically significant (χ²(1, N = 50) = 5.41, p = 0.020, φ = 0.33), indicating a moderate effect size and supporting the role of maternal inflammation as a predictor of neonatal morbidity. Overall neonatal survival to discharge was 94% (n = 47), reflecting favorable outcomes with appropriate perinatal care.

Maternal serum IL-6 concentrations demonstrated a significant inverse correlation with gestational age at delivery (*r* = −0.71, p < 0.001), as illustrated in Figure 1. Women who delivered before 32 weeks exhibited markedly higher IL-6 levels compared to those with later preterm births. This relationship supports the hypothesis that heightened systemic inflammation contributes to the premature onset of labor and reduced fetal maturity.

## Discussion

This prospective study confirmed that elevated maternal IL-6 concentrations are strongly associated with preterm delivery and adverse neonatal outcomes. These results are consistent with findings by Romero et al. [6-8], who demonstrated that IL-6 in amniotic fluid is a reliable marker of intrauterine inflammation and microbial invasion. Similarly, Chaemsaithong et al. [9,12] and Tsiartas et al. [11] reported elevated IL-6 in women delivering before 34 weeks, underscoring its predictive value.

The pathophysiologic basis for these associations lies in the pro-inflammatory role of IL-6, which promotes prostaglandin synthesis, cervical ripening, and myometrial contractility [5,14,16]. Increased IL-6 production by decidual macrophages and fetal membranes amplifies cytokine signaling, leading to matrix metalloproteinase activation and premature membrane weakening [13,17]. Even in the absence of infection, this sterile inflammation can prematurely trigger labor, a mechanism increasingly recognized in perinatal medicine [18].

Recent reviews highlight that elevated IL-6, particularly in maternal serum, represents a systemic reflection of localized intrauterine immune activation [15,19]. Kacerovsky and Jacobsson [15] demonstrated that cytokine elevation precedes preterm birth even in sterile cases, while Jung et al. [20] linked IL-6-associated fetal inflammatory response with neonatal sepsis and poor perinatal adaptation.

Clinically, our findings reinforce the diagnostic potential of IL-6 as a biomarker for preterm labor. In settings like regional Romania, where resources are limited, IL-6 assessment could guide early intervention, such as corticosteroid administration or referral to tertiary neonatal units. The correlation between elevated IL-6 and NICU admissions in our cohort further supports its prognostic utility.

The significant χ² association between elevated maternal IL-6 levels and NICU admission (χ²(1, N = 50) = 5.41, p = 0.020, φ = 0.33) reinforces the pathogenic role of intrauterine inflammation in preterm birth and neonatal morbidity. This finding supports IL-6 as a clinically relevant biomarker capable of identifying pregnancies at increased risk of neonatal compromise.

Although this study demonstrated a significant association between maternal IL-6 levels and preterm delivery, certain mechanistic aspects remain beyond its scope. In particular, the lack of parallel analysis of placental histopathology, amniotic fluid cytokine profiles, or microbiologic cultures limits our ability to directly differentiate between infectious and sterile inflammatory etiologies. These procedures were not performed due to ethical and clinical constraints associated with invasive sampling during preterm labor. Future research will aim to incorporate such correlational analyses in a larger cohort to strengthen the understanding of IL-6-mediated pathways leading to preterm birth.

Limitations of this study include its modest sample size and single-center design, which may limit generalizability. Additionally, amniotic fluid IL-6 levels were not measured, preventing direct comparison between systemic and local inflammatory responses. Future multicenter research combining multiple inflammatory markers (IL-6, IL-8, CRP, TNF-α) with clinical risk factors could improve predictive accuracy.

## Conclusions

Maternal serum IL-6 levels correlated inversely with gestational age and neonatal birthweight and were significantly elevated in women delivering before 32 weeks of gestation. These findings reinforce the role of IL-6 as a reliable biomarker of intrauterine inflammation and preterm birth risk. Incorporating IL-6 testing into the evaluation of women presenting with threatened preterm labor may improve early identification of high-risk pregnancies and guide timely interventions. Further multicenter studies with larger sample sizes are warranted to validate these results and establish standardized IL-6 thresholds for clinical use.
